# The intralaminar thalamus: a review of its role as a target in functional neurosurgery

**DOI:** 10.1093/braincomms/fcad003

**Published:** 2023-02-02

**Authors:** Hisse Arnts, Stan E Coolen, Filipe Wolff Fernandes, Rick Schuurman, Joachim K Krauss, Henk J Groenewegen, Pepijn van den Munckhof

**Affiliations:** Department of Neurosurgery, Amsterdam University Medical Centers, location Academic Medical Center, Amsterdam, The Netherlands; Department of Neurosurgery, Radboud University Medical Center, Nijmegen, The Netherlands; Department of Neurosurgery, Amsterdam University Medical Centers, location Academic Medical Center, Amsterdam, The Netherlands; Department of Neurosurgery, Hannover Medical School, Hannover, Germany; Department of Neurosurgery, Amsterdam University Medical Centers, location Academic Medical Center, Amsterdam, The Netherlands; Department of Neurosurgery, Hannover Medical School, Hannover, Germany; Department of Anatomy and Neurosciences, Neuroscience Campus Amsterdam, Amsterdam University Medical Centers, location VU University Medical Center, Amsterdam, The Netherlands; Department of Neurosurgery, Amsterdam University Medical Centers, location Academic Medical Center, Amsterdam, The Netherlands

**Keywords:** deep brain stimulation, ablation, thalamus, neurological disorders, psychiatric disorders

## Abstract

The intralaminar thalamus, in particular the centromedian-parafascicular complex, forms a strategic node between ascending information from the spinal cord and brainstem and forebrain circuitry that involves the cerebral cortex and basal ganglia. A large body of evidence shows that this functionally heterogeneous region regulates information transmission in different cortical circuits, and is involved in a variety of functions, including cognition, arousal, consciousness and processing of pain signals. Not surprisingly, the intralaminar thalamus has been a target area for (radio)surgical ablation and deep brain stimulation (DBS) in different neurological and psychiatric disorders. Historically, ablation and stimulation of the intralaminar thalamus have been explored in patients with pain, epilepsy and Tourette syndrome. Moreover, DBS has been used as an experimental treatment for disorders of consciousness and a variety of movement disorders. In this review, we provide a comprehensive analysis of the underlying mechanisms of stimulation and ablation of the intralaminar nuclei, historical clinical evidence, and more recent (experimental) studies in animals and humans to define the present and future role of the intralaminar thalamus as a target in the treatment of neurological and psychiatric disorders.

## Teaser

Arnts et al. report that the intralaminar thalamus is a strategic node within different basal ganglia–thalamocortical networks. This thin strip of nuclei forms an important target for stereotactic ablation and neurostimulation procedures to modulate disease networks in a variety of disorders, including pain, epilepsy, Tourette, Parkinson’s and disorders of consciousness.

## Graphical Abstract

**Graphical Abstract:**
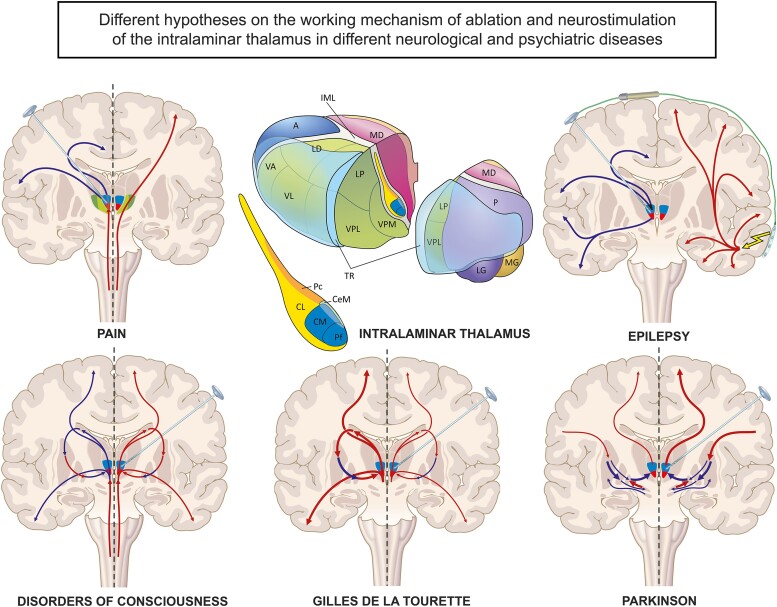

## Introduction

The intralaminar part of the thalamus, through its extensive connections with the striatum and widespread cortical targets, is critically involved in a variety of cognitive functions, including memory, attention, and perception.^1-5^ Moreover, it remains a vital structure in the relay of nociceptive input to the cerebral cortex.^6^ Not surprisingly, the intralaminar thalamus, in particular the centromedian-parafascicular (CM-Pf) complex, has long been an area of interest as a target in functional neurosurgery, including deep brain stimulation (DBS) procedures and (radio)surgical ablation to influence disease processes in various neurological and psychiatric disorders, such as pain, epilepsy, Gilles de la Tourette syndrome, movement disorders and disorders of consciousness (DOC) following traumatic brain injury. In this review, we provide a comprehensive analysis of historical evidence and more recent (experimental) studies in both animals and humans in order to define the present and future role of the intralaminar thalamus as a target in functional neurosurgery.

## Brief anatomy of the intralaminar thalamus

The intralaminar thalamic nuclei are embedded in a thin lamina of myelinated fibres (lamina medullaris interna) that courses centrally through the thalamus along its rostro-caudal axis. The intralaminar complex consists of a variety of nuclei that are classically subdivided into a rostral (anterior) and a caudal (posterior) group.^7-9^ Traditionally, the intralaminar thalamus, together with the midline thalamic nuclei, has been viewed as a non-specific relay of ascending information projecting diffusely to the cortex. However, with the advent of more sophisticated neuroanatomical tracer methods, it has been demonstrated that distinct intralaminar thalamic nuclei influence specific cortical areas, striatal regions, and also parts of the pallidum and subthalamic nucleus that form relays of closed re-entrant cortical–basal ganglia loops.^7-10^ This provides the intralaminar thalamus with a strong modulatory influence on functionally distinct cortical–basal ganglia circuits.^8^ One aspect of the connectivity of the intralaminar nuclei that still characterize them as ‘non-specific’ is the brainstem input from monoaminergic (locus coeruleus), serotonergic (raphe nuclei), and cholinergic cell groups (dorsolateral tegmental nucleus), the pedunculopontine nucleus and neurons in the reticular formation that distribute over the entire intralaminar complex (for a complete overview, see Krout et al.).^11^ Such inputs may thus jointly affect extensive cortical areas and multiple cortical–subcortical loop systems.^8,11^ Nevertheless, inputs from several brainstem and spinal cord pain-relaying nuclei, cerebellum, basal forebrain, and several basal ganglia structures show a clear selectivity in their intralaminar targets.

**Fig. 1:**
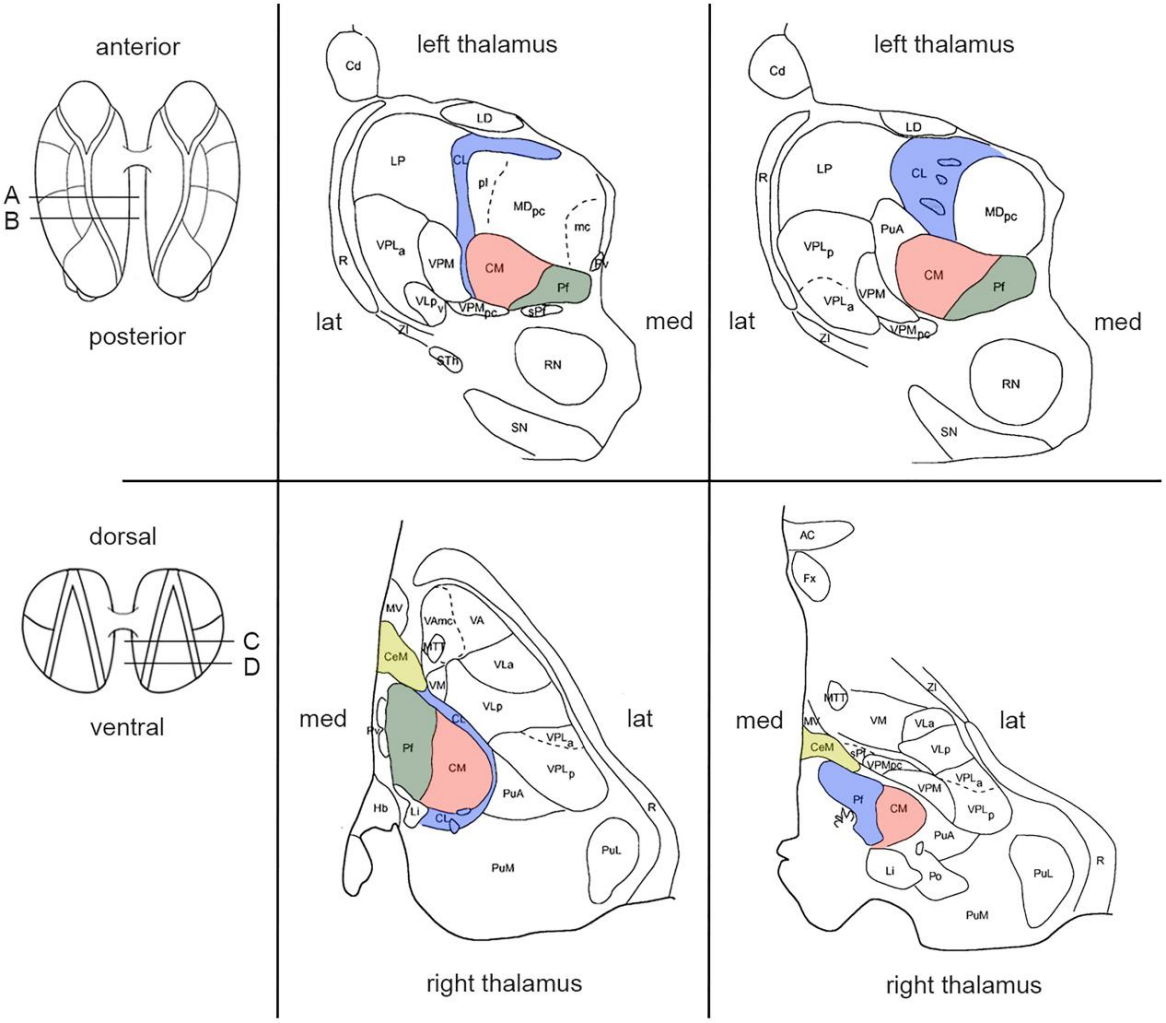
**Schematic anatomy of the intralaminar thalamic nuclei adapted from Morel’s stereotactic atlas of the human thalamus.^12^** (Note that there is a variety in nomenclature and competing parcellations of the human thalamus between different stereotactic atlases.^13^) (**A**) Coronal plate perpendicular to the intercommissural plane, 7.2 mm anterior to the posterior commissure. (**B**) Coronal plate perpendicular to the intercommissural plane, 5.4 mm anterior to the posterior commissure. (**C**) Axial section oriented parallel to the AC-PC plane, 2.7 mls dorsal to the intercommissural plane. (**D**) Axial section oriented parallel to the AC-PC plane at the height of the intercommissural plane. Abbreviations: AC = anterior commissure, Cd = caudate nucleus, CeM = central medial nucleus, CL = central lateral nucleus, CM = centromedian nucleus, Fx = fornix, Hb = habenular nucleus, lat. = lateral, LD = lateral dorsal nucleus, Li = limitans nucleus, LP = lateral posterior nucleus, MDmc = mediodorsal nucleus, magnocellular division, MDpc = mediodorsal nucleus, parvocellular division, med. = medial, MTT = mammillothalamic tract, MV = medioventral nucleus, Pf = *parafascicular nucleus*, Po = posterior nucleus, PuA = anterior pulvinar, PuL = lateral pulvinar, PuM = medial pulvinar, Pv = paraventricular nuclei, R = reticular thalamic nucleus, RN = red nucleus, SN = substantia nigra, sPf = subparafascicular nucleus, STh = subthalamic nucleus, VAmc = ventral anterior nucleus, magnocellular division, VLa = ventral lateral anterior nucleus, VM = ventral medial nucleus, VPLa = ventral posterior lateral nucleus, anterior division, VLp = ventral lateral posterior nucleus, VPLp = ventral posterior lateral nucleus, posterior division, VPMpc = ventral posterior medial nucleus, parvocellular division, ZI = zona incerta.

**Fig. 2:**
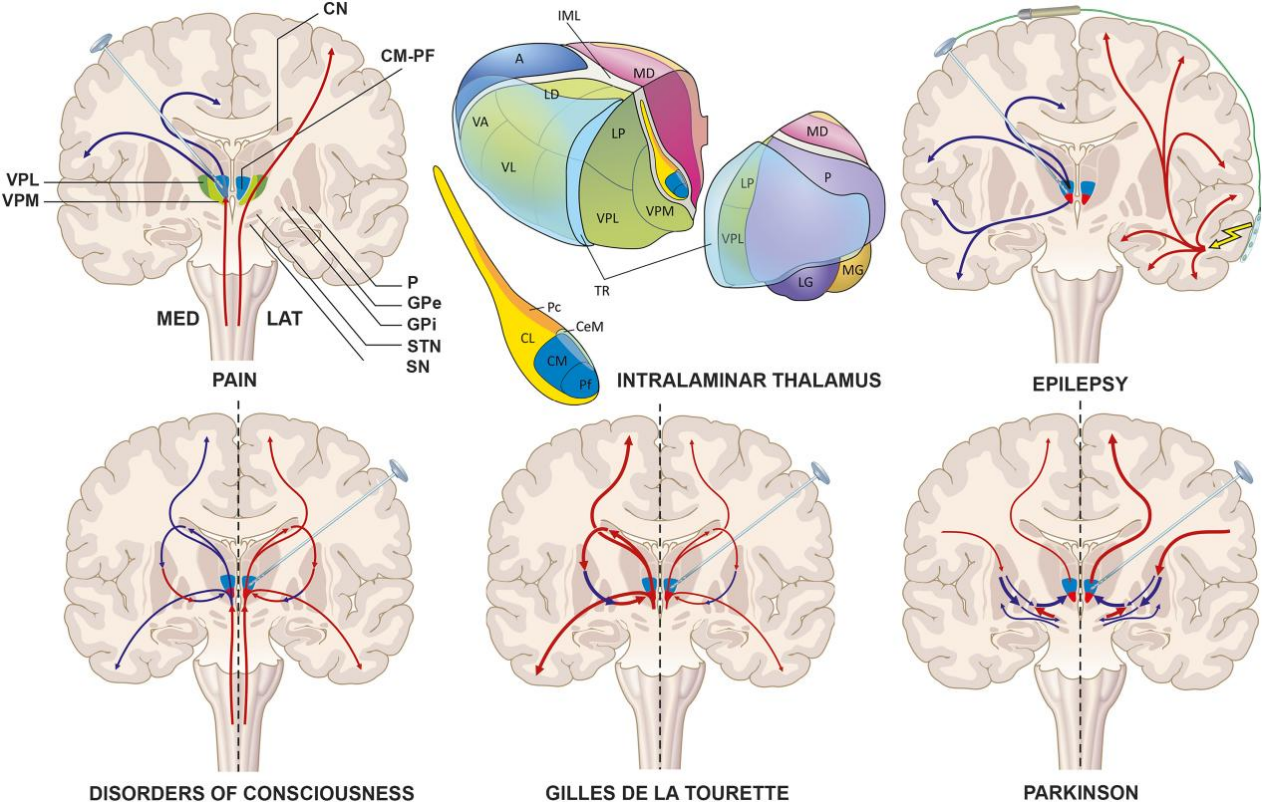
**Different (hypothetical) working-mechanisms of ablation/neurostimulation of the intralaminar thalamus in a coronal slice through the brain and basal ganglia.** Upper left image: Course of the medial and lateral pain pathways, in which the medial pain pathway travels through the CM-Pf of the intralaminar thalamus and ascending pain signals are suppressed by neurostimulation. Upper right image: Responsive-neurostimulation in epilepsy in which signs of epilepsy are registered by a cortical strip lead that directly activates the neurostimulator to send an ‘epilepsy blocking’ intralaminar signal through the brain electrode. Lower left image: DBS in DOC. On the left hemisphere: a situation in which loss of striatal output results in a negative-feedback loop and excessive (pallidal) inhibition of the thalamus. On the right hemisphere: a situation in which this negative-feedback loop is breached by neurostimulation. Lower middle image: DBS in GTS. On the left hemisphere: a situation in which striatal ‘overactivity’ eventually results in inhibition of the GPi and excitation of the thalamus and, thereby, excessive (sub)cortical activity. On the right hemisphere: a situation in which intralaminar neurostimulation inhibits this excessive overactivity. Lower right image: DBS in Parkinson’s disease. On the left hemisphere: classical model of Parkinson’s disease, in which there is excessive inhibition of the thalamus by the GPi, secondary to a decreased excitatory drive from the STN and decreased inhibitory input from the putamen. On the right hemisphere: a situation in which neurostimulation restores this aberrant thalamic outflow. Upper middle image: Schematic 3D drawing of the relationship of the individual intralaminar nuclei (also see Mai et al.).^13^ Abbreviations in 2D-image: CN = caudate nucleus, CM-Pf = centromedian-parafascicular complex, GPE = globus pallidus externus, GPI = globus pallidus internus, P = putamen, SN = substantia nigra, STN = subthalamic nucleus, VPM = ventral posteromedial nucleus, VPL = ventral posterolateral nucleus. Abbreviations in 3D-image: A = anterior thalamic nucleus, CeM = central medial nucleus, CL = central lateral nucleus, IML = lamina medullaris interna, LD = lateral dorsal nucleus, LG = lateral geniculate nucleus, LP = lateral posterior nucleus, MD = mediodorsal nucleus, MG = medial geniculate nucleus, Pc = paracentral nucleus, VA = ventral anterior nucleus, VL = ventral lateral nucleus, P = pulvinar, TR = thalamic reticular nucleus. Image made by Inge Kos.

As indicated above, the intralaminar thalamic nuclei can be subdivided into a rostral and a caudal group. The rostral group includes the central lateral nucleus (CL), the paracentral nucleus (Pc) and the central medial nucleus (CeM). The caudal group corresponds to the CM-Pf, the ‘central complex’. In humans, the CM is relatively large compared with the other intralaminar nuclei and measures about 10 mm in diameter. This relatively large size of the human CM compared with the other intralaminar nuclei is related to the great expansion in the course of the evolutionary development of neocortical and associated striatal areas with which CM is connected. Together with the CL, the CM-Pf complex constitutes the major part of the intralaminar nuclei (see Figs. 1 and 2). The rostral intralaminar nuclei receive ascending subcortical input from the above-mentioned transmitter-specific brainstem nuclei as well as, more specifically, from the spino- and trigeminothalamic tracts (pain-conducting pathways) and the cerebellum. Cortically, CL is mainly interconnected with motor and parietal cortical areas, while CeM and Pc have more intense interconnections with prefrontal and medial limbic cortical areas, including the anterior cingulate cortex.^5,7^ Subcortically, the rostral group predominantly projects to the striatum, in particular the central parts of the caudate-putamen. Likewise, the caudal nuclei receive rich inputs from a large part of the brainstem, especially from the ascending reticular activating system. The CM and, though to a lesser extent, the Pf also receive robust projections from the internal segment of the globus pallidus and the reticular part of the substantia nigra, i.e. the output nuclei of the basal ganglia.^2^ Cortically, the CM is predominantly connected to sensorimotor cortical areas, while the Pf has important interconnections with the prefrontal and cingulate cortices. Similar to the rostral intralaminar nuclei, the caudal group projects strongly to the striatum.^5^ Within the striatum, CM preferentially targets the dorsolateral caudate and putamen (i.e. the recipient domain of sensorimotor cortical areas), while the Pf mainly projects to cognitive and limbic areas (i.e. central caudate and putamen). Further projections, although less strong, have been described to the pallidal complex and subthalamic nucleus.^2,14^ Though the connectivity of each of the individual intralaminar nuclei presented above remains primarily based on evidence from experimental animal studies in rats and subhuman primates, a recent human study using diffusion tensor tractography largely confirms these observations.^15,16^ In conclusion, the intralaminar thalamic nuclei are strongly influenced by monoaminergic, cholinergic, serotonergic and reticular arousal systems in the brainstem, as well as more differentially by ascending pain pathways and outputs from cerebellum and basal ganglia. With their strong projections to basal ganglia targets and reciprocal connections with the cerebral cortex, these thalamic nuclei seem to have a crucial position to modulate the functioning of a variety of cortical–subcortical circuits in the sensorimotor, attentional, cognitive and emotional domains.

### A short note on the composition of the axonal fibre tracts related to the intralaminar complex

An important aspect of the anatomy of the intralaminar thalamus in the context of neurosurgical ablations and DBS is the composition, course and orientation of fibre tracts that traverse or connect these nuclei with other brain regions. As stated above, the individual intralaminar nuclei are embedded in the lamina medullaris interna of the thalamus that, in neuroanatomical fibre stains, clearly shows to be composed of myelinated fibres. However, the relatively strong inputs from brainstem areas, such as those originating from the monoaminergic, serotonergic and cholinergic nuclei are thin and non-myelinated. Furthermore, these ascending fibre tracts are oriented in a caudal to rostral direction. Thus, fibres that are destined for the rostral (anterior) intralaminar nuclei course close to or through the caudal (posterior) intralaminar nuclei. Neurosurgical lesions or stimulation of the caudal nuclei, therefore, may affect fibre projections to the more rostral intralaminar nuclei as well. Similarly, the CM-Pf complex, in its medial aspects, is traversed by the fasciculus retroflexus, which connects the habenular complex in the medial (epi)thalamus with the interpeduncular nucleus and the raphe nuclei in the midbrain. This fibre tract is an important direct and indirect regulator of the serotonergic and cholinergic systems in the brainstem and may be, indirectly, affected by neurosurgical procedures aimed at the CM-Pf.

### Anatomy and nomenclature of the human thalamus

As indicated above, data on the connections of the intralaminar thalamic nuclei are primarily based on observations in non-human primates and rodents. It should further be noted that anatomical descriptions and delineations of individual nuclei of the human thalamus, including the intralaminar complex, have been rather heterogeneous.^13^ As a consequence, this is an important limitation in the interpretation of results of neurosurgical procedures reported below that rely on information from different stereotactic atlases. The delineations of the intralaminar thalamic nuclei as presented in Figs. 1 and 2 are primarily based on the stereotactic atlas of Morel et al.^12^ and have different names and representations in other atlases. Moreover, stereotactic coordinates of individual nuclei, including those of the CL and CM-Pf, vary greatly between studies and are often patient-specific, since the anatomical location of the intralaminar thalamus has a strong relation to the variable width of the third ventricle. A greater consensus on the delineations, anatomical terminology, and use of standardized stereotactic reference points of the thalamic nuclei would be of importance for better comparability of future neurosurgical approaches and clinical results.^13^

## The intralaminar thalamus as target for pain

### Rationale

The CL and CM-Pf are involved in pain perception and receive signals from the spinothalamic tract that originate from neurons in the deep dorsal horn of the spinal cord and the spinal trigeminal nucleus, as well as, multisynaptically, from pain-relaying areas in the reticular formation of the brainstem.^17^ Classically, two pain-conducting systems have been distinguished: the ‘medial pain system’, with its relay in the intralaminar thalamus, and the ‘lateral pain system’, with its relay in the ventral posterolateral (VPL) and posteromedial nucleus (VPM) of the thalamus. Different dimensions of pain perception have been associated with these two systems and are, classically, attributed to specific structures within the thalamus.^18,19^ The medial pain system was previously thought to be mainly associated with affective dimensions of pain, including feelings of unpleasantness and emotions associated with future implications of pain. The fact that the pain-related output of the intralaminar thalamus is mainly directed towards the cingulate gyrus, an important interface for the interaction between pain and emotions, contributes to this view. Pain, however, is a highly complex phenomenon for which this distinction between two systems appears to be too simple. Modern views of pain consider the various cerebral cortical and thalamic structures primarily as part of networks or matrices which are dynamically involved in different aspects of the perception of pain. It is now thought that the pathway via the medial and intralaminar thalamus to limbic structures encodes affective aspects of pain that converge with discriminative information processed via other thalamocortical pathways. Others have even argued that the sensory and affective dimensions of pain are inseparable aspects of a unitary experience, and pain itself is a unidimensional construct.^20^ Certainly, the strict dichotomy used previously is an oversimplification, since sensory-discriminative and limbic brain regions may also be sensitive to cognitive processes.

In the neural circuits for pain, the nuclei of the intralaminar thalamus function as a relay, that has previously been assumed to act in the defense against ‘nociceptive aggression’.^19,21^ These nuclei are thought to maintain a gate control function, propagating only salient noxious stimuli and suppressing certain other stimuli. This gate control function might be disturbed in patients with specific pain syndromes, such as deafferentation pain, which is known to present with sustained neuronal bursting or ‘hyperactivity’ in the intralaminar thalamic nuclei.^22,23^ Opioid analgesics, such as morphine have long been known to inhibit (evoked) activity in the medial and intralaminar thalamus.^19,24^ This formed part of the scientific basis for the early stereotactic ablation and stimulation studies of the medial structures of the thalamus for the relief of various pain syndromes. In general, both ablation and high-frequency DBS are thought to interfere with aberrant activity in the intralaminar thalamus in patients with a variety of pain syndromes (see Fig. 2).

### Ablation of the intralaminar thalamus in pain

In the early years of functional stereotactic surgery, Hécaen et al.^25^ reported on a successful thalamotomy for intractable pain, primarily targeting the CM-Pf complex of the thalamus. Furthermore, isolated stereotactic ablation of the CM for chronic pain was performed by Talairach^26^ as early as 1949 and later by Monnier and Fischer.^27^ Soon thereafter, ‘medial thalamotomies’ became a treatment option in patients with a wide variety of pain syndromes. Different case series were reported with various targets, commonly including the CM-Pf complex and, less often, the CL (for an overview, see Supplementary Table).^17,28,29^ Inconsistent use of terminology and clinical jargon along with inaccuracies in target descriptions, however, limits the interpretation of these early studies. In most cases, medial thalamotomies encompassed multiple targets, as well as areas of the medial pain pathways caudal to the intralaminar nuclei. Moreover, it often remains unclear which exact pain syndrome was treated. Since medial thalamotomies were considered relatively safe, the procedure remained particularly appealing in the early beginnings of functional neurosurgery. Generally, medial thalamotomies were associated with a low rate of neurologic complications, most often consisting of minor paresthesia, and transient cognitive disturbances.

Different case-series in the early literature report a rather encouraging, but widely varying, direct pain relief in patients after medial thalamotomies, ranging from 13 to 100%. In a large overview of the early literature, Tasker^30^ described the results of medial thalamotomies in a total of 175 patients with nociceptive (usually cancer) pain. Overall pain relief was described in 46% of patients. In contrast, in patients with neuropathic pain, relief was present in only 29%, suggesting that the procedure might be more effective in patients with nociceptive pain. Jeanmonod and colleagues^31^ reported a higher rate (67%) of patients attaining pain relief (50–100%) in their series of 45 patients who underwent medial thalamotomy (with the CL as the main target). Dougherty et al.^32^ reviewed data from 34 publications with a total of 913 patients and indicated that initial partial pain relief was found in 73% of patients with a variety of pain syndromes.

In addition to surgical ablation of the medial thalamus, radiosurgical ablation was also studied in different types of cancer pain, trigeminal neuralgia, thalamic pain and phantom limb pain. Leksell^33^ was the first to perform a medial thalamotomy for the treatment of intractable pain by the use of radiosurgery in 1972. The CM-Pf complex was targeted in a total of 25 patients. Of these, 10 demonstrated improvement of pain. Hereafter, several series of gamma knife thalamotomy for pain were published. In the case-series of Steiner et al.^34^ two-thirds of their 52 patients experienced early pain relief with different response rates of 56–67%, confirming the efficacy of the procedure. Moreover, Young et al.^35,36^ described an efficacy rate (≥50% pain reduction) of 53% in various patients with intractable pain. Based on the cumulative results of these series, a potential success rate of up to 60% was quoted, with a complication rate of 6–17%. Follow-up studies, however, demonstrated relatively high recurrence rates of 30–45% after radiosurgical ablation and up to 88% after surgical ablation.^30,37,38^

### DBS of the intralaminar thalamus in pain

In comparison with ablative surgery, DBS of the intralaminar thalamus has been used less often. Most early animal and human studies of intracranial stimulation for pain relief focused on other targets, such as the periaqueductal grey (PAG).^39^ DBS of the CM-Pf was pioneered by Ray and Burton^40^ and by Andy^41^ in the late 1970s. Remarkably, Ray and Burton^40^ described pain relief of 50% or more in 21 of 28 patients with different pain syndromes. In 2001, Krauss et al.^42^ described the preliminary results of CM-Pf DBS in 11 patients with neuropathic pain and showed that the short-term effects of CM-Pf stimulation were superior to more commonly used targets for pain, such as the VPL/VPM. Since then, there has been re-interest in the CM-Pf as a target for chronic pain. In 2016, Sims-Williams and colleagues^43^ demonstrated that CM-Pf DBS was comparable to PAG stimulation in facial pain associated with anaesthesia dolorosa. More recently, the long-term follow-up of 40 patients with various neuropathic pain syndromes who underwent both implantations of DBS electrodes in the CM-Pf and VPL/VPM was published. Of the total of 20 patients that were treated with CM-Pf DBS over the course of 17 years, including those that were published by Krauss et al.^42^ in 2001, half of them showed an average improvement of ≥50% in pain intensity. There was no difference when comparing the efficacy of CM-Pf versus VPL/VPM DBS. Though follow-up times varied, these recent results are the first evidence of the long-term effectiveness of CM-Pf DBS in various types of neuropathic pain.

### The future role of the intralaminar thalamus as a target for pain relief

The renewed interest in the CM-Pf and CL for the treatment of a variety of pain syndromes, especially in the context of ablation, may accelerate in the coming years with the advent of alternative non-invasive ablation techniques, such as high-intensity focused ultrasound.^17,44-46^ For now, there is no consensus on when to perform ablation or DBS of the intralaminar thalamus, which of the intralaminar and medial thalamic nuclei constitutes the optimal target, and which patients might be suitable candidates. Moreover, ablation and DBS might have various effects on pain and different efficacy in different pain syndromes.^28,30,47^ Larger studies with longer follow-up times that compare ablation and/or DBS of the intralaminar thalamus with other intracranial targets, such as the VPL/VPM and PAG, remain necessary. Moreover, fundamental and clinical research is needed to determine how ablation and stimulation of the intralaminar thalamus affect activity in other pain-encoding structures, as well as how these techniques induce changes at a network level.

## The intralaminar thalamus as target in epilepsy

### Rationale

Despite optimal pharmacological treatment, around one-third of patients with epilepsy have drug-resistant epilepsy (DRE) and suffer from uncontrollable focal and/or generalized seizures.^48,49^ Historically, different surgical treatment options were explored for patients with DRE, including functional hemispherectomy, lobectomy, and corpus callosotomy. Some became a standard treatment for patients with specific forms of epilepsy, such as corticectomy for dysplasia, and a variety of resection methods for the temporal lobe and its surrounding structures, including the amygdala and hippocampus.^50,51^ However, resective neurosurgery remains limited in patients with seizures that arise from more than one brain location, from eloquent regions, or in those with epilepsy that is generalized in onset. For such patients, a wide variety of other neurosurgical techniques have been investigated, including ablation and stimulation of different brain structures.^52^

Both animal and human studies examined the role of the intralaminar thalamus in the propagation and control of epileptic seizures, resulting in different hypotheses on the role of the intralaminar thalamus in epilepsy. Some studies suggested that the intralaminar thalamus itself is involved in the process of seizure-initiation.^53,54^ For instance, early EEG and electrical stimulation studies in patients with epilepsy showed that activation of the intralaminar thalamus, especially the CM, is associated with EEG signs characteristic for generalized epilepsy and typical absence-like attacks, suggesting a strong relationship between the occurrence of intralaminar (over)activity with epilepsy.^54^ More recent functional MRI studies confirm this relationship between intralaminar activity and (idiopathic) generalized epilepsy, especially for the CM-Pf complex.^55^ Other structures of the intralaminar thalamus are also thought to have a role in the propagation and spread of seizures in different types of epilepsy. For instance, animal studies showed that lesions of the CL can disrupt the generation of experimentally induced absence seizures.^56,57^ Also, pharmacological activation of the Pf was reported to suppress signs of absence epilepsy, and electrical stimulation interrupted focal hippocampal seizures in mesial temporal lobe epilepsy.^58,59^ The intralaminar thalamus might also indirectly control seizures, by regulating the excitability of other structures.^60,61^ It is thought that the intralaminar thalamus controls the threshold of seizures through a combination of inhibitory GABA-mediated neurotransmission and excitatory (glutamate) input.^54,59^ Not surprisingly, disruptions in the balance between inhibitory (GABA) and excitatory (glutamate) neuronal activity are a pathological feature of many epilepsy syndromes.^62-65^

Though the exact working mechanism of ablation and DBS of the intralaminar thalamus remains unknown, it is generally thought that both methods can block the genesis or propagation of seizures by (local) inhibition of activity (see Fig. 2).^66^ Previous studies showed that high-frequency DBS is most effective in treating epilepsy and may restore neurotransmitter disbalance and concomitant normal regulatory control of the thalamus.^54,67^ For instance, Nanda et al.^68^ showed that CM-DBS raises GABA levels in the striatum of awake rhesus monkeys. Furthermore, Fernández-Cabrera et al.^69^ showed that CM-DBS lowers pathological levels of glutamatergic activity in the prefrontal cortex in rats.

### Ablation of the intralaminar thalamus in epilepsy

Only a few animal studies reported on the effects of (chemically induced) lesions of the intralaminar thalamus in various forms of epilepsy.^57,70^ Moreover, there is only casuistic early evidence of the use of stereotactic ablation of the intralaminar thalamus in humans. These early studies are difficult to interpret, since lesions of the intralaminar thalamus, especially in the region of the CM, were always accompanied by a wide variety of lesions to other thalamic structures. Most previous lesion studies in humans have reported on the effects of stereotactic ablation of other parts of the thalamus, especially the anterior nucleus. However, a recent study on radiofrequency ablation of the CM in six patients by Aguado-Carillo et al.^71^ reported a 79–98% reduction in the number of generalized seizures. While this report showed that the procedure is effective and safe, longer follow-up times are necessary to assess the long-term effects of CM ablation and to compare these with the effects of neurostimulation.

### DBS of the intralaminar thalamus in epilepsy

DBS for epilepsy has a rich history and has been performed on multiple brain targets. Though the earliest reports on intracranial neurostimulation in humans involved cerebellar structures, the thalamus quickly became an important area of interest. In the 1980s, around 50 years after Penfield proposed the CM as a potential target for the treatment of epilepsy, the group of Velasco et al.^72^ conducted the first open-label study on CM-DBS in five patients with generalized epilepsy. Previously, they had assessed the safety and efficacy of CM-DBS in various animal models.^54^ At three months follow-up, a reduction of 80–100% was reported in generalized tonic–clonic seizures (GTC) and a 60–100% reduction in complex partial seizures. Moreover, all patients experienced a seizure reduction of at least 50% and were considered responders to treatment. In response to these promising results, Fisher et al.^73^ performed a cross-over study of CM-DBS in seven patients to further assess feasibility and safety in 1992 (for an overview, see Supplementary Table). In a subsequent study by Velasco^74^ in 23 patients with various seizure patterns, CM-DBS resulted in a significant decrease in the total number of seizures at three months follow-up. Patients with GTC seemed one of the strongest responders, with 89% reduction of seizures per month. In contrast, patients with other seizure patterns, such as those with Lennox-Gastaut syndrome (LGS) showed no significant reduction after DBS, suggesting a specific role for CM-DBS in different types of seizures/epilepsy. Similarly, variable results for different types of epilepsy were reported in a further study with longer follow-up, leading to the conclusion that CM-DBS would be more beneficial for patients with generalized tonic–clonic seizures than other seizure types or syndromes. However, after these early explorations, later studies showed contrasting results. For instance, in 2000, Velasco^54^ showed that CM-DBS for Lennox-Gastaut resulted in an average seizure reduction of 82%, which is in sharp contrast to their earlier report. Another study in 13 other LGS-patients showed a similar average in seizure reduction after CM-DBS (80%).^75^ Also, in 2009 Cukiert et al.^76^ reported a 98% seizure reduction after CM-DBS in one of their patients with LGS after a mean period of 1.5 years follow-up.

There is a relative paucity in the literature on CM-DBS since 2009, possibly as a result of the effects of the SANTE-trial: a large randomized controlled trial of DBS of the anterior nucleus of the thalamus (ANT) (see below). After this period, Valentin et al.^77^ reported the results of a randomized CM-DBS cross-over trial in 11 patients suffering from either generalized or frontal lobe epilepsy. The average reduction in seizure frequency was around 80% among patients with generalized epilepsy and around 20% among those with frontal lobe epilepsy. Furthermore, a few other studies have been published on the effects of CM-DBS in a variety of seizure types and epilepsy syndromes.^78,79^ Most recently, Cukiert et al.^80^ published the results of a prospective open-label study on the efficacy of high-frequency CM-DBS in 20 patients with generalized epilepsy. After a median follow-up time of >2.5 years, 90% of patients were considered responders (≥50% seizure frequency reduction), and one patient achieved seizure freedom. Also, the results of a study investigating the efficacy of CM-DBS (*n* = 5) and combined CM and ANT-DBS (*n* = 11) in 16 children and adults suffering from DRE were reported.^81^ After a median follow-up time of 80 months, 63% of patients responded to stimulation (≥50% reduction in seizures) with a median seizure frequency reduction of 58%. Interestingly, median seizure frequency reduction and responder rate did not differ between the CM and the CM/ANT group.

### The future role of the intralaminar thalamus as a target in control of epilepsy

Currently, besides the thalamus, several other structures are examined as targets for DBS, including the subthalamic nucleus, posterior hypothalamus, hippocampus, cerebellum, caudate nucleus, corpus callosum and brainstem regions.^82^ Nevertheless, the thalamus remains the most important target for the treatment of DRE in clinical practice. In addition to the CM, the ANT has long been a target of interest and subject of many animal and human studies. In fact, ANT-DBS is the only FDA-approved treatment of DRE, which was granted in response to the long-term follow-up results of the SANTE-trail, the first large randomized controlled cross-over DBS trial in 110 patients with epilepsy.^83,84^ In the SANTE-trial, a 56% median percent reduction in seizure frequency was reported in patients with epilepsy after two years of follow-up. This further improved to 69% with a responder rate (≥50% reduction in seizure frequency) of 68% at five years. The ANT is now the most widely used target for DBS in the treatment of DRE and, not surprisingly, receives the most scientific interest. However, no randomized clinical trial has ever compared the efficacy of ANT-DBS and CM-DBS in treating seizures in DRE-patients. Consequently, there is no evidence of superiority or difference in the efficacy of ANT-DBS or CM-DBS in the treatment of different forms of DRE. The heterogeneity of patient groups and varieties in stimulation paradigms and parameters (for instance, low- versus high-frequency DBS) also limits the comparison between the two targets using previous evidence. For now, CM-DBS is still used in a selection of patients with different seizure types in a variety of neurosurgical centres and might be a reasonable alternative to ANT-DBS in non-responders.

## The intralaminar thalamus as target in Gilles de la Tourette syndrome

### Rationale

Gilles de la Tourette Syndrome (GTS) is a severe neurological disorder characterized by multiple motor or vocal tics, usually accompanied by a variety of other psychopathological disorders, such as attention deficit hyperactivity disorder, obsessive–compulsive behaviour, depression and anxiety. Several lines of biochemical, imaging, neurophysiological and genetic research indicate that various relays in basal ganglia–thalamocortical circuits play a crucial role in the pathophysiology of GTS.^85-87^ In general, it is thought that GTS is caused by a failure of inhibition within basal ganglia–thalamocortical loops and abnormal signalling of neurotransmitters, such as dopamine and GABA. Specifically, it is thought that subsets of projection neurons become active in inappropriate contexts as a result of dysfunctional inhibitory GABA interneurons within the striatum.^88,89^ This results in disinhibition of thalamocortical projections and increased excitability of both motor and limbic regions, leading to the inappropriate expression of sensory and motor phenomena, i.e. tics.

Usually, patients with GTS are treated with a combination of behavioural therapies, pharmacological interventions, and, if necessary, invasive non-surgical treatment, such as botulinum toxin injections. In patients with severe GTS that are refractory to these interventions, functional neurosurgery may be a treatment option.^90-92^ Both ablation and DBS are known measures to interfere with aberrant signalling in the basal ganglia–thalamocortical network of GTS patients and are thought to reduce (pre)motor and limbic excitability.^93^ DBS of the intralaminar thalamus in GTS has been associated with a decrease in the release of striatal dopamine.^94,95^ This suggests that DBS might actively inhibit striatal activity through dopaminergic modulation, consequently counteracting the abnormal activity in the basal ganglia that would lead to inefficient impulse control (see Fig. 2).^96^ Furthermore, improvement of both motor and vocal tics after DBS may be associated with the correction of abnormal structural connectivity within specific thalamocortical (pre)motor pathways.^97^ However, the exact mechanism by which ablation or DBS controls tics in GTS has yet to be further explored.

### Ablation of the intralaminar thalamus in GTS

Since the 1960s, there have been several reports on surgical procedures targeting a wide variety of brain structures for the treatment of GTS and other tic disorders.^90,91^ In 1970, Hassler and Dieckmann^98,99^ were the first to publish their results on stereotactic thalamotomy in three patients with severe GTS. All three patients had bilateral lesions in the medial thalamus, rostral intralaminar thalamus and, in one patient with facial tics, also the nucleus ventro-oralis internus (Voi). Motor and vocal tics were reduced by, respectively, 100%, 90% and 70%. A few years later, the same authors reported on further findings in their patients. Eventually, a total of nine patients with GTS were treated. Of them, three underwent unilateral and six bilateral ablations of the intralaminar and medial thalamus. A 50–100% reduction in tic frequency was reported. Three of the patients who had been treated bilaterally were even reported to have tic reductions of 90–100%. In 1987, Cappabianca et al.^100^ published the long-term results of thalamotomy in a cluster of four patients, targeting the mediodorsal and intralaminar thalamus. While one patient experienced almost complete remission of symptoms, the results in the other three were relatively limited. In 2001, Babel et al.^101^ published a series of 16 patients with treatment-resistant GTS. Six of these patients were targeted in the intralaminar thalamus (CM) in addition to the zona incerta and/or the ventrolateral thalamus. After a mean follow-up of 12 years, an average reduction of 61% in vocal tics and 69% in motor tics were observed. From a contemporary viewpoint, the results of several early ablative studies are difficult to appreciate, since most of them were limited by a lack of quantifying outcome or specifying the assessments by which quantification was performed. With the advent of DBS for GTS in the early 2000s, ablative procedures, though considered relatively safe, were gradually abandoned.

### DBS of the intralaminar thalamus in GTS

In 1999, Vandewalle et al.^102^ published the first study on DBS in a patient with GTS, targeting the border zone between CM, the substantia periventricularis (Spv) and the Voi as outlined previously in the work of Hassler and Dieckmann. After one year, stimulation was sufficient to abolish all tics in their patient. Following this observation, a follow-up study on bilateral CM-Spv-Voi stimulation in two other GTS-patients was published in 2003.^103^ Once again, all major vocal and motor tics disappeared after the procedure. Eventually, after a mean follow-up of 27 months, an average reduction of 82% in tics was reported. Thereafter, several studies were published on the effects of thalamic, but also on pallidal DBS in GTS.^92,104^ While some groups continued to use the original Hassler/Dieckmann target, the exact thalamic target remained unclear in other studies, with some authors indicating that the primary target was the CM or CM-Pf.^105^ Servello et al.^106^ reported on a large cohort of patients treated with DBS, targeting the CM-Pf-VO in 34 patients with GTS. The average Yale Global Tic Severity Score (YGTSS) scores of the 19 patients who reached 2-year follow-up decreased from a pre-operative average of 77 (out of 100) to 37, indicating a significant 52% reduction in tics and disease-related impairment. In 18 of these patients, scores even further decreased from an average of 81 pre-operatively to 22 at the five to six-year follow-up (reduction of 73%).^107^ Several other studies since then have reported quite variable outcomes, ranging anywhere from nearly no improvement in the primary tic-related outcome, to complete remission of tics.^92,104,105,108-110^ A 2016 meta-analysis showed that DBS of thalamic structures, in general, compared over all previous studies with a total of 78 patients, resulted in an average reduction of around 39% in tics.^104^ However, in addition to the intralaminar thalamus, various other brain targets have been explored in patients with GTS, including the (posteroventral lateral and anteromedial) globus pallidus internus (Gpi), nucleus accumbens, ventral caudate, anterior internal capsule, globus pallidus externus (Gpe) and subthalamic nucleus (STN).^91,104^ The meta-analysis showed that the anteromedial Gpi may potentially be a slightly more effective target than the intralaminar thalamus, with an average tic reduction in reported literature of 47% after DBS, though these results were not significant.^104^ Further evidence from a large international database and registry, containing a total of 185 patients, reported no significant differences between CM-Pf DBS versus Gpi DBS (46 versus 51% reduction in YGTSS at 12 months follow-up). Finally, a recent prospective randomized double-blind sham-controlled study on a series of 10 patients who underwent implantation of both CM-Pf and Gpi DBS electrodes showed that, at group level, Gpi but not thalamic DBS resulted in a significant tic reduction compared to baseline.^111^ During long-term follow-up (mean 90 months after surgery), there was no improvement of tics, comorbidities, and quality of life at the group level, however, single patients benefitted continuously from thalamic DBS. Remarkably, at that time 50% of patients had discontinued DBS. Thus, there is still an ongoing debate on the most effective brain target controlling GTS.^111,112^

### Future role of the intralaminar thalamus in the treatment of GTS

A broad range of selected targets is studied in experimental settings in specialized centers for functional neurosurgery. The procedure is generally well-tolerated in both the paediatric as well as adult population with relatively minor complications or adverse events.^104,113^ While the intralaminar thalamus has been a region of interest since the earliest days of DBS for GTS, in recent years the focus seems to have shifted towards Gpi stimulation.^104^ However, there is still no consensus on which is the better target. Moreover, the efficacy of DBS in GTS patients of different age groups remains questionable.^92,113^ A recent systematic review of GTS-DBS in the paediatric population revealed that, after exclusion of the most severe quartile of GTS-patients, thalamic DBS was significantly more effective than Gpi DBS in reducing tic severity.^113^ Future studies remain necessary to determine which target and stimulation paradigm is best suitable for which patient.^114,115^

## The intralaminar thalamus as target for restoring consciousness after severe brain injury

### Rationale

Neurons within the intralaminar thalamus are well known to have a primary role in maintaining arousal and wakefulness. Early animal studies already revealed that the intralaminar thalamus is part of an important ascending arousal pathway, involved in producing broad cortical ‘awakening responses’ after stimulation. The intralaminar thalamus receives ascending input from different brainstem arousal systems, including afferents from the mesencephalic reticular formation, locus coeruleus, dorsolateral tegmental and pedunculopontine nuclei, but also from basal forebrain regions that are involved in arousal.^9,116^ Since the intralaminar thalamus has strong reciprocal connections with several regions of the frontal cortex, posterior cortical association areas that support poly-sensory integration, and the basal ganglia, it is crucially positioned to play a key role in arousal regulation.^9,116^ Evidence from animal and human studies shows that variations of activity within the intralaminar thalamus are associated with changes in, for instance, behavioural alertness, attention, working-memory performance, and transitions during the normal sleep–wake cycle.^116-119^ Therefore, it is thought that the intralaminar thalamus acts as an important regulator of arousal during wakeful states and that its activity changes in response to specific task demands.^3,4,120,121^

Damage to the intralaminar thalamus is associated with a wide variety of deficits, including impaired attentional processing, working-memory problems and hypersomnolence.^122,123^ Severe brain injury is associated with damage or ‘inactivation’ of neurons within the intralaminar thalamus, which, in severe cases, produces DOC. DOC may be a temporary phase after severe brain injury, or a more permanent state if patients fail to recover. Distinct clinical DOC syndromes have been identified following the acute comatose phase after brain damage, such as the unresponsive wakefulness syndrome (UWS), a condition of unresponsiveness in the presence of wakefulness (previously known as apallic syndrome, coma vigil or the vegetative state), and the minimally conscious state (MCS), a state characterized by partial preservation of consciousness with reproducible signs of minimal awareness.^122,124^ With the advancement of mechanical ventilation in the 1950s, more patients with severe brain damage survived their initial injuries and concomitantly developed chronic forms of DOC. A rise in the incidence of these patients with the severest forms of brain injury prompted early clinical investigators to explore the use of neurostimulation for the restoration of arousal.^125^

The intralaminar thalamus has, from the beginning, become the most important target for these DBS studies. Early and more recent animal studies showed the potential of neurostimulation of the intralaminar thalamus to produce both arousal responses, such as signs of cognitive enhancement, including sustained attention, as well as increased behavioural performance.^121,126-128^ In humans, it is thought that partial loss of neurons within the thalamus after brain damage results in a concomitant loss of thalamocortical and thalamostriatal activity.^129^ In resting conditions, the tonic firing of Gpi neurons inhibits the thalamocortical system, including the intralaminar thalamic nuclei.^129,130^ Normally, corticostriatal activity, by inhibiting Gpi neurons, disinhibits thalamocortical activity. However, the loss of thalamostriatal and thalamocortical activity, due to damage of thalamic neurons, might create a negative-feedback loop resulting in a loss of disinhibition of the thalamus via the GPi. Consequently, the activity of the intralaminar nuclei is further reduced causing further down-regulation of global brain dynamics. DBS of the intralaminar thalamus and return of local activity within the intralaminar thalamus may reverse this aberrant cascade of signals and facilitate restoration of arousal regulation (see Fig. 2).

### Ablation of the intralaminar thalamus in disorders of consciousness

Since prolonged DOC are usually caused by widespread damage to the brain or more focal damage to arousal structures, inducing extra lesions for functional recovery has been considered rather paradoxical. Not surprisingly, experimental studies in patients with DOC have focused on stimulation rather than ablation of key structures within the human arousal system. However, a recent report on the use of thalamic low-intensity focused ultrasound has suggested that this may cause some temporary behavioural improvement in patients with MCS.^131^

### Stimulation of the intralaminar thalamus for disorders of consciousness

Clinical investigations on the use of functional neurosurgery for the restoration of consciousness in patients with severe brain injury started as early as in the late 1960s and 1970s.^125,132^ In 1968, McLardey et al.^133^ were the first to perform DBS in a young patient with UWS after severe brain injury, targeting both the CM-Pf and the mesencephalic reticular formation. Though their temporary form of neurostimulation was accompanied by significant neurophysiological ‘arousal-effects’, little clinical signs of improvement were reported. Hereafter, a couple of other attempts were taken to restore consciousness through temporary DBS of other brain structures, including the GPi and more anteromedial areas of the thalamus, all with little sustained effects on consciousness (for an overview, see Supplementary Table).^134-136^ After these early attempts, a larger group of patients was treated with DBS throughout the 1990s in different centers throughout Europe, the United States and Japan. Three separate studies reported the effects of unilateral CM-Pf DBS in a total of around 50 patients.^137-139^ The majority of patients were shown to have an acute behavioural arousal response with DBS, associated with consistent physiological responses, desynchronization of the EEG, and increased cerebral metabolic rates measured by PET. Moreover, a significant proportion of patients was reported to have a return of oral feeding and showed some signs of environmental awareness. Some research groups involved in the trial even reported that a small number of patients with traumatic brain injury showed a significant functional improvement, including the recovery of independence.^138,139^ However, later studies criticized that, in retrospect, these patients already showed signs of minimal consciousness before DBS, and received their surgery within a 3–6 months post-injury interval, which is well within the window for spontaneous recovery after severe brain injury.^126,139,140^ Besides, since these studies were performed in heterogeneous groups of patients with different aetiologies, without the use of standardized assessment scales of consciousness, and with various follow-up times, it is difficult to interpret and generalize findings from these studies.

After these heterogeneous results of early DBS studies, Schiff et al.^141^ first proposed to perform DBS in patients with MCS instead of UWS, since MCS patients might have more intact functional brain networks and, therefore, a larger capacity for functional recovery. After careful animal studies, and extensive ethical deliberations, they reported improvements after DBS of the CL in one MCS patient who suffered traumatic brain injury six years before the intervention.^140,141^ DBS restored communication and various behavioural items on different subscales of the Coma Recovery Scale (CRS-R), an internationally accepted scale for assessing consciousness in patients with severe brain injury. However, no longer follow-up results have been reported. Moreover, this patient remained a single subject from a series of three, of which the other two showed no such spectacular signs of improvements after stimulation.^142^ In reaction, similar attempts followed in different European countries. For instance, Magrassi et al.^143^ performed CM-Pf DBS in three patients. Though they showed limited improvements of the CRS-R score in all patients, none of them returned to a fully conscious state or showed consistent signs of communication. In a larger series, Chudy et al.^144^ reported that three out of their 14 UWS and MCS patients regained a fully conscious state after CM-Pf DBS and recovery of independence. These three patients were all diagnosed with MCS. However, these patients were treated within the window for spontaneous recovery after severe brain injury. Finally, Lemaire et al.^145^ reported the results of CM-Pf DBS in five patients with severe traumatic brain injury (4 MCS and 1 UWS). Two of their patients (1 UWS and 1 MCS) showed improvements on different subscales of the CRS-R score after DBS, as well as an increased cerebral metabolic rate measured by PET. However, neither of these patients regained a fully conscious state.

Throughout history, various stimulation settings have been used in studies of DBS for DOC, including a large variation in stimulation pulse frequency and amplitude, which may be one of the many factors that explain the heterogeneity of the effects of stimulation.^132^ Recently, Arnts et al. have shown that, in DOC, both low- and high-frequency stimulation can increase arousal, though low-frequency stimulation, often accompanied by a larger volume of tissue activation in the intralaminar thalamus, is associated with increased functional connectivity and direct return of arousal.^146^ This observation is somewhat in contrast with evidence from animal research, in which (very) low-frequency stimulation seems to result in a decrease in arousal and behaviour.^121^

In recent years, researchers have further begun to explore the therapeutic potential of DBS for other patient categories with less severe brain injuries, including those without DOC, but with severe cognitive impairment.^147^ It remains a question whether DBS in these patients, as well as performing the procedure relatively early after injury, can induce return of residual brain functions and/or prevent secondary complications after brain injury.^144,148^

### Future of the intralaminar thalamus in the treatment of disorders of consciousness

The idea of stimulation of the intralaminar thalamus for improvement of consciousness has a relative ‘enigmatic’ history with convincing fundamental theories from animal studies, but heterogeneous clinical effects in patients with severe brain injury. Nevertheless, cases have been reported with spectacular effects on arousal and wakefulness. The majority of studies are characterized by methodological limitations and are difficult to generalize, because of the large heterogeneity of patients concerning aetiology and their pre-operative level of consciousness. Up until today, large double-blind studies are lacking.^125^ Research in patients with DOC remains challenging, mainly because of ethical issues, scarcity of suitable candidates, and heterogeneity of patient groups. For now, DBS is only performed in an explorative setting in a couple of highly specialized neurosurgical centres throughout the world. It forms part of a broader arsenal of experimental neuromodulatory techniques that are used in patients with DOC, including vagal nerve stimulation, and more non-invasive stimulation techniques, such as focused ultrasound, transcranial magnetic stimulation and transcranial direct-current stimulation.^149,150^ Future research in larger, more homogeneous groups of patients is necessary to determine the risk–benefit ratio for performing DBS in individual patients with severe brain injury.

## The intralaminar thalamus as target for Parkinson’s disease

### Rationale

As described above, the intralaminar thalamus is strongly involved in the basal ganglia thalamocortical circuitry and has reciprocal connections with several regions of the frontal cortex that are engaged in the planning and execution of movement, including the supplementary motor area and the anterior cingulate cortex. Not surprisingly, the intralaminar thalamus has been the subject of research in patients with movement disorders, especially in patients with Parkinson’s disease (PD).^151,152^ The dopamine deficiency in PD leads to reduced inhibition of the indirect pathway and reduced excitation of the direct pathway, resulting in overactive neuronal discharges of the GPi and subthalamic nucleus (STN), which increases the inhibition of thalamocortical and brainstem motor systems.^153,154^ This eventually interferes with motor execution and is thought to be the basis for the characteristic clinical features of PD, including rigidity and bradykinesia. In addition, there is considerable evidence from animal and human studies that the intralaminar thalamus, in particular the caudal nuclei, also has a role in the pathophysiology of PD. For instance, autopsy studies in patients with PD showed the presence of a profound neuronal loss in the intralaminar thalamus, especially in the CM-Pf, but to a lesser extent also in the CL.^155,156^ Moreover, in animals treated with MPTP injections, a chemical neurotoxin that destroys dopaminergic neurons in the substantia nigra, neuronal cell loss develops in the CM-Pf.^157^ Furthermore, metabolic studies showed reactive changes in the activity of CM-Pf neurons projecting to both the STN and the striatum in PD, further indicating an intricate role for the CM-Pf in the development of specific impairments in PD.^158-161^

The traditional targets in functional neurosurgery for PD patients whose response to drug therapy is poor or unsatisfactory have long been the GPi and the STN.^162^ Since the CM-Pf receives strong projections from the GPi and sends a strong (glutamatergic) output to the STN, it has been suggested as an alternative target to study in patients with PD.^151,160^ Various animal and human studies were performed, investigating the role of CM-Pf ablation and DBS for a variety of PD symptoms, including the treatment of L-dopa induced dyskinesias, tremor and other symptoms, such as freezing of gait. Besides, CM-Pf DBS has further been investigated as a treatment for other hyperkinetic movement disorders not primarily associated with PD, such as (essential) tremor and other dyskinesias.^161,163,164^

### Ablation of the intralaminar thalamus in Parkinson’s disease

The first suggestion that the CM might have a critical role in movement disorders was made by Schulman et al.^165^ in 1957. A few years later, Rand et al.^166^ reported favourable results in PD patients after lesions of the CM. Hereafter, Adams and Rutkin^167^ were the first to perform a larger study in a group of 26 patients with both unilateral (*n* = 9) and bilateral (*n* = 17) PD. After a follow-up of >3 months, those that received CM ablation for unilateral PD showed a good or even excellent result, indicating complete relief or significant improvement of rigidity with a return to normal activity without motor impairments. In contrast, a satisfactory result, but not as much improvement was seen in patients with bilateral PD. In 1984, however, Vasin et al.^168^ reported less favourable effects of CM ablation in 15 patients with PD and severe akinesia. A satisfactory therapeutic effect with a marked improvement of motor impairments was seen in only three patients. Five other patients showed some motor improvements without improvement in overall motor activity and two had an increase in akinesia to nearly complete immobility after surgery. In 1996, Jeanmonod et al.^169^ reported the effects of stereotactic medial thalamotomies (mainly targeting the CL and CM-Pf) in 22 patients with a variety of movement disorders, including patients with PD. Though a 50–100% relief of symptoms, such as tremor, rigidity and bradykinesia was reported in 43% of all patients, there was only a fairly limited decrease in the mean UPDRS motor subscore. After these early explorations, little evidence exists for the ablation of the intralaminar thalamus in movement disorders in humans. The remainder of the evidence seems to exist of anecdotal descriptions of secondary lesions after DBS attempts, which sometimes causes some (minor) improvements of PD symptoms (for an overview, see Supplementary Table).

### DBS of the intralaminar thalamus for the treatment of Parkinson’s disease

Early electrostimulation studies already suggested that stimulation of the CM is associated with general activation of movements, a simultaneous decline in muscle tonus, and reduction of tremor, and might therefore be valuable in patients with movement disorders. Most early evidence on the use of CM-Pf DBS for movement disorders is, however, indirectly derived from the study of patients with pain disorders. In 1980, Andy et al.^41^ described the results of DBS of the CM-Pf complex in three patients with treatment-refractory pain and concomitant movement disorders. In these patients, CM-Pf DBS not only resulted in the decrease of pain, but also in improvement of rigidity, spasticity and cervical dystonia. In 2002, Krauss et al.^164^ described similar results in a study of CM-Pf DBS for refractory pain. In their prospective study of 12 patients, three with additional movement disorders showed signs of improvement after DBS. Furthermore, a retrospective analysis of large studies on thalamic DBS for PD, which was performed around that period, reported that patients with active DBS leads in the region of the CM-Pf might have a better response than those with DBS leads limited to the ventral intermediate nucleus. This increased the interest for clinical studies on the use and efficacy of CM-Pf in PD.^170^

In response to these findings, Mazzone et al.^171^ performed an explorative study on the additional use of CM-Pf DBS in combination with more widely used DBS targets for PD in 2006. Six patients received bilateral DBS of both the GPi as well as the CM-Pf complex. In all patients, a significant improvement of UPDRS motor subscores was achieved by simultaneous activation of both targets. GPi DBS produced a mean reduction in UPDRS motor subscore of 42%, while CM-Pf DBS only produced a mean reduction of 35%, though with a slightly better effect on freezing of gait. Combined stimulation of both the GPi and CM-Pf resulted in a mean reduction of around 50%. Therefore, the authors suggested further research on the additional use of CM-Pf stimulation in PD patients. Subsequently, the same group of researchers reported on their experience using a multi-target approach for PD, combining CM-Pf DBS with conventional STN-DBS. In their two patients, STN-DBS remained far better in improving UPDRS scores than CM-Pf DBS, though CM-Pf DBS was shown to have a more profound effect on tremor and therefore seemed to somewhat complement STN-DBS. A further compilation of their long-term experience using different multi-target approaches for DBS, including combined CM-Pf and GPi/STN-DBS, also showed a similar additional effect on tremor and motor UPDRS subscores, though this effect diminished over the longer term.^151,172^ After these explorative studies, there is a rather abrupt paucity in the study of CM-Pf DBS for PD, possibly because of a shift in focus towards other targets, including the pedunculopontine nucleus, which has well-known connections with the Pf.^151,173^ Since then, the literature on CM-Pf for movement disorders is limited to a single case-study for a patient with essential tremor.^163^

### Future of the intralaminar thalamus for the treatment of Parkinson’s disease

DBS of the CM-Pf might be a possible ‘add-on’ in the treatment of specific symptoms that are resistant to DBS of more established targets, including the STN and GPi.^151^ Evidence from studies in humans confirms that ablation or CM-Pf DBS alone is not a suitable strategy for the treatment of PD symptoms. Moreover, there seems to be too limited evidence to make any conclusions about the use of a multi-target strategy that includes the CM-Pf complex in PD. While some reports clearly show that CM-Pf DBS has beneficial effects on tremor and sometimes results in a reduction of dyskinesias, these salient effects might also depend on the spread of current to other thalamic targets. For now, there is a consensus that STN and GPi are the most effective DBS targets for the treatment of the key motor symptoms of PD. Nevertheless, the CM-Pf remains a structure of interest, since new evidence shows that it might be associated with a variety of non-motor symptoms that are inherent to different movement disorders.^157^

## Discussion

The intralaminar thalamus, consisting of a densely populated area of nuclei, has long served as an important target in functional neurosurgery for various neurological disorders. A wide variety of both animal and human studies have been performed from the early start of stereotactic neurosurgery, the results of which are sometimes difficult to interpret because of heterogeneous use of thalamic nomenclature, limited study follow-up, and the fact that lesions or DBS often involve multiple nuclei or even different parts of the thalamus. Though the intralaminar thalamus was once thought to represent ‘a forgotten component of the great loop of connections joining the cerebral cortex via the basal ganglia’ it is regaining renewed attention.^174,175^ As outlined by the present review, ablation and DBS of different components of the intralaminar thalamus have long been studied in the treatment of pain, epilepsy, GTS, DOC, and a variety of movement disorders. Throughout the years, different hypotheses have been developed about the working mechanism of ablation and stimulation in these disorders (see Fig. 2). However, functional neurosurgery of the intralaminar thalamus remains still underexplored and, as of yet, has not become a clinical standard in most of the above-described conditions. Nevertheless, the intralaminar thalamus still attracts ongoing attention. For instance, there is a resurrection of interest in the use of CM-Pf and CL as targets for both ablation and DBS in the treatment of pain. In particular, the intralaminar thalamus is attractive with the advent of less-invasive lesion techniques in combination with the fact that it is considered a safe zone for these interventions with few side effects. Moreover, recent long-term follow-up results of CM-Pf DBS show that the target may be a useful treatment option in addition to, for instance, the somatosensory thalamus in selected patients with severe and medically refractory neuropathic pain. The CM-Pf complex also remains an important target in the treatment of epilepsy and may still be a useful alternative target in addition to the FDA-approved ANT. Randomized controlled trials that compare both targets for the control of generalized and focal forms of epilepsy are much awaited and remain necessary to make any conclusions about superiority between the two targets. The role of thalamic DBS in the treatment of GTS also needs further evaluation, in particular with regard to GPi DBS. For now, CM-Pf DBS or CL DBS is also still considered experimental in DOC, since therapeutic evidence of restoration of consciousness and purposeful behaviour after severe brain injury remains limited. With promising evidence from animal studies, further exploration in patients with disturbances in arousal, cognition or memory will undoubtedly follow in the coming years.^121,128^ Finally, CM-Pf DBS seems to have a limited role in the treatment of movement disorders including PD. However, its use in these patients has, once again, returned to the background, most likely because of a combination of limited results and increasing benefits of DBS of more conventional targets. Future research will reveal if CM-Pf DBS is effective as an additional treatment for the associative functional deficits that are often inherent to movement disorders and remain somewhat resistant to the more established targets.

The fact that stimulation and ablation of the intralaminar thalamus has beneficial effects in different neurological and psychiatric diseases implies the coexistence of different ablation/stimulation-dependent mechanisms on well-known thalamocortical and thalamostrial loops. From a ‘connectomic’ point of view, the intralaminar thalamus may be viewed as an integrative hub within different, more distant and less densely connected brain networks.^176^ It seems to form a ‘funnel’ that integrates and spatially compresses information stemming from widespread brain regions. This explains why a single lesion or electrode in this small area of the brain can modulate different malfunctioning large-scale brain networks and, hypothetically, induce different (sub)cortical effects in various neurological and psychiatric disorders (as illustrated in Fig. 2). Though, for some indications, functional neurosurgery of the intralaminar thalamus seems to lead to significant therapeutic benefit at the group level, there still remains a large heterogeneity in the effects of ablation and stimulation in individual patients.^177^ Some authors have proposed that these variations in effect may be explained by the possibility that pathological brain networks are patient-specific and may vary between patients with the same disorder.^176^ By using new neurophysiological and neuroimaging tools, it may be possible to acquire more information about a patient’s individual disease-network profile. Information about a patient’s individual ‘symptom-network’ may eventually tailor which ‘funnel’ to target with functional neurosurgery. For which specific symptom(s) the intralaminar thalamus will be the most suitable funnel, remains a matter of research.

## Conclusion

There is a relatively large body of early and more recent evidence that functional neurosurgery, including ablation and DBS procedures, can offer beneficial effects for patients with treatment-refractory pain, epilepsy and GTS. However, there is no clinical consensus for the use of intralaminar thalamic nuclei in various other disorders as compared to more established targets, although comparative research would be much awaited. For several disorders, functional neurosurgery of the intralaminar thalamus is still evidently experimental, such as in patients with severe brain injury. Future research on the anatomy, physiology and pathophysiology of the intralaminar thalamus and its relation to large-scale (malfunctioning) brain networks in different neurological and psychiatric disorders will allow for a more evidence-based approach for using the intralaminar thalamus in functional neurosurgery. With the increasing accessibility of DBS and less-invasive stereotactic techniques in different centers throughout the world, as well as the expanding use of new neurophysiological and neuroimaging techniques, more insights into this enigmatic brain region will be gained rather sooner than later.

## Supplementary Material

fcad003_Supplementary_Data

## Data Availability

Data sharing is not applicable to this review article as no new data were created or analysed in this study.

## Abbreviations

ANT =: anterior nucleus of the thalamus
CeM =: central medial nucleus
CM =: centromedian nucleus
CM-Pf =: centromedian-parafascicular complex
CL =: central lateral nucleus
CRS-R =: Coma Recovery Scale-Revised
DBS =: deep brain stimulation
DOC =: disorders of consciousness
DRE =: drug-resistant epilepsy
GABA =: gamma-aminobutyric acid
GTS =: Gilles de la Tourette Syndrome
GTC =: generalized tonic–clonic seizures
GPe =: globus pallidus externus
GPi =: globus pallidus internus
LGS =: Lennox-Gastaut syndrome
MCS =: minimally conscious state
PAG =: periaqueductal grey
PD =: Parkinson’s disease
Pf =: parafascicular nucleus
Spv =: substantia periventricularis
STN =: subthalamic nucleus
UPDRS =: Unified Parkinson’s Disease Rating Scale
UWS =: unresponsive wakefulness syndrome
Voi =: nucleus ventro-oralis internus
VPL =: ventral posterolateral nucleus
VPM =: ventral posteromedial nucleus
YGTSS =: Yale Global Tic Severity Score
