# Supplementary material for: The intralaminar thalamus: a review of its role as a target in functional neurosurgery
Source: Brain Commun. 2023 Feb 2;5(3):fcad003. doi: 10.1093/braincomms/fcad003 (PMC10244065; doi:10.1093/braincomms/fcad003)
Supplement: fcad003_Supplementary_Data [file fcad003_supplementary_data.pdf]

SUPPLEMENTARY MATERIAL: STUDIES ON ABLATION AND DBS OF THE INTRALAMINAR NUCLEI OF THE THALAMUS

1. STUDIES ON ABLATION/DBS OF THE INTRALAMINAR THALAMUS IN PAIN

1.1 Ablation (for review of early studies <1980 on ablation for pain, see Tasker et al.)

Year	Authors	Type of patients	Type of ablation	n (% male)	Target	Follow-up	Results
1980	Steiner et al.	Cancer pain	Gamma Knife	52 (N/A%)	CM-Pf	1 to >36 months	Good pain relief (8), moderate pain relief (18), no significant pain relief (24).
1982[1]	Niizuma et al.	Central (thalamic) pain (17), PLP (1)	Lesion electrode	18 (78%)	CM	9 months-6 years	Effective in 56% of patients: 5/18 complete relief of pain, 5/18 patients partial relief of pain (limited duration of pain relief: half a year at the longest).
1993	Jeanmonod et al.	Various (neurogenic pain)	Lesion electrode	45 (N/A%)	Medial thalamus	±14 months	67% of patients reached 50-100% pain relief. 29% of patients had complete relief of pain.
1995	Young et al.	Spinal disorders (10), central (thalamic) pain (3), PHN (3), FP (1), other (3)	Gamma Knife	20 (N/A%)	Medial thalamus	1-22 months	65% of patients experienced excellent or good pain relief.
1995	Hariz & Bergenheim	Central (thalamic) pain (5), cancer pain (2), FP (2)	Lesion electrode	9 (N/A%)	CM	±16 months	3/5 patients with central (thalamic) pain, 1/2 with cancer pain, and 1/2 with FP had beneficial results (≥ 50% reduction in pain).
1996[2]/2001	Young et al.	Various (intractable pain and FP)	Gamma Knife	61 (N/A%)	IL	±74 months	32/61 patients (53%) achieved a ≥50% reduction in visual analog scale/pain scores.

2001[3]	Jeanmonod et al.	Various (chronic peripheral/central neurogenic pain)	Lesion electrode	96 (N/A%)	CL	±45 months	53% of patients benefited from a relief ≥50%. Complete relief in 19%.
2006	Keep et al.	Central (thalamic) pain	Gamma Knife	1 (100%)	CM	7 years	Significant improvement in pain without need for medication.
2018	Urgosik et al.	Different types of TP (23) central (thalamic) pain (4), PLP (1), other (2)	Gamma Knife	30 (33%)	CM-Pf	±24 months	13/30 (43%) had an initial successful result (pain intensity ≤50%) with 1 of these patients being completely pain-free. Pain recurred in 4 patients (31%) after 24 months.
2020	Franzini et al.	TP (4), posttraumatic cord/roots (2), PHN (1), other (1)	Gamma Knife	8 (50%)	CL	±24 months	At the last follow-up visits, 5/8 patients reported ≥ 50% VAS pain reduction. Mean VAS-score significantly reduced after 24 months. Pain had recurred in 2 patients.
2020	Gallay et al.	TP	Focused ultrasound	8 (63%)	CL	±53 months	Mean pain relief: 51% at 3 months, 71% at 1 year and 78% at the longest follow-up. This represents 63% good outcomes (pain relief ≥ 50%) at 3 months, 88% at 1 year and 100% at last follow-up.
Legend: *CM = centromedian nucleus; CM-Pf = centromedian-parafascicular complex; CL = central lateral nucleus; FP = facial pain; IL = intralaminar nuclei; N/A = Not Available; PHN = post-herpetic neuralgia; PLP = phantom limb pain; TP = trigeminal pain.*							

1.2 DBS

Year	Authors	Type of patients	n (% male)	Target	Stimulation parameters	Follow-up	Results

1976[4]	Boethius et al.	Post-traumatic cord/roots (1), PLP (1), FP (2), other (1)	5 (N/A%)	CM-Pf (5) (including other targets)	10-100 Hz / 0.15-7 mA / 0.1-0.3 ms	6 months (1), 6 weeks (4)	Pain-free (40%), reduced dysesthesia (40%), no relief (20%).
1979[5]	Thoden et al.	Chronic intractable pain (N/A)	7 (N/A%)	CM-Pf (and parts of MD)	10-100 Hz / Variable amplitude / 50 - 350 µs	N/A	(Pain reducing) paresthesia's in contralateral half of body and the area of pain.
1980	Ray & Burton	FBS (11), Cancer pain (6), post-traumatic cord/roots (5), FP (2), thalamic stroke (1), PLP (1), other (2)	28 (54%)	CM-Pf	N/A	±14 months	75% experienced ≥50% reduction in pain. In FBS 64% of patients, cancer pain 100%, post-traumatic cord/roots 80%, FP 50%, thalamic stroke 100%, PLP 0%, and other 100% effect.
1980	Andy	Pain associated with dyskinesia's	3 (33%)	CM-Pf	25-125 Hz / 6-20 V / 0.1-0.5 ms	>3 months (1), N/A (2)	Total pain relief (2), sufficient pain relief (1).
1983[6]	Andy	Thalamic stroke (2), post-traumatic cord/roots (1), other (2)	5 (40%)	CM-Pf/other IL	50 Hz / 0.1-5.0 V / 200 µs	≥ 12 months (1), ≥ 7 months (1), N/A (3)	Total relief of pain (3), episodes of recurring pain (1), controllable pain (1).
1995	Hariz & Bergenheim	Post-traumatic cord/roots (2), FP (1), thalamic stroke (1)	4 (N/A%)	CM	N/A	±16 months	2 of 4 (with post-traumatic cord/roots and thalamic stroke) had successful pain relief (≥ 50% reduction in pain).
2001[7]/ 2002	Krauss et al.	Chronic neuropathic pain (N/A)	12 (50%)	CM-Pf	130 Hz / variable amplitude / 180-210 µs	N/A	Significant improvement in pain scores relative to pre-operative conditions (not further specified).
2016	Sims-Williams et al.	TP, anaesthesia dolorosa	3 (33%)	CM-Pf and PAG	70-150 Hz for CM-Pf / 5-10 Hz for PAG	19 ± 8 months	Reduction in mean pain score by 56% with PAG and 67% with CM-Pf stimulation.
2017[8]	Hollingworth	TP, anaesthesia dolorosa (1), PLP (1), post-stroke (1)	3 (N/A%)	CM-Pf and PAG	128-132 Hz for CM-Pf / 10 Hz for PAG / 3,5-4,5mA	3 years	Improvement of mean VAS scores and return to daily activities.

2021	Abdallat et al. (follow-up Krauss et al.)	(Thalamic) stroke/hemorrhage (4), herpes zoster (3), trauma (4), other (9)	20 (50%)	CM-Pf	131 Hz / 2.5 V / 210 µs	±63 months; range 3-180 months	Improvement of VAS average pain score by ≥50% in 50% of patients at last follow-up and ≥30% in 65% of patients.
Legend: *CM = centromedian nucleus; CM-Pf = centromedian-parafascicular complex; FBS = failed back surgery; FP = facial pain; IL = intralaminar nuclei; MD = medial dorsal nucleus; N/A = Not Available; PLP = phantom limb pain; TP = trigeminal pain.*							

2. STUDIES ON ABLATION/DBS OF THE INTRALAMINAR THALAMUS IN EPILEPSY

2.1 Ablation (lesion electrode)

Year	Authors	n (% male)	Type of epilepsy/seizure	Target	Follow-up	Results
2021	Aguado-Carillo et al.	6 (33%)	Primarily GE (6) of which LGS (1), or MFE (1)	CM	±28 months	Reduction of 79-98% in GS. Focal aware seizures remained unchanged.
Legend: *CM = centromedian nucleus; GE = generalized epilepsy; GS = generalized seizures; LGS = Lennox-Gastaut syndrome; MFE = multifocal epilepsy;*						

2.2 DBS

Year	Authors	n (% male)	Type of epilepsy/seizure	Target	Stimulation parameters	Follow-up	Results
1987[9]	Velasco et al.	5 (60%)	TC and CP	CM	60-100 Hz / 0.8-2.0 mA / 0.1 ms	3 months	80-100% reduction in TC, 60-100% reduction in CP.

1992	Fisher et al.	7 (43%)	TC (5) of which LGS (2), TS (1), and CP (1)	CM	65 Hz / 0.5-10 V / 90 µs	9-month cross-over phase and long-term open-label period of ±8 months.	≥50% improvement in GS in >50% of patients.
1993	Velasco et al.	23 (61%)	TC (9), PM (3), CP (5), LGS (6)	CM	60 Hz / 8-15 V (400-1250 µA) / 1.0 ms	9-month cross-over	Significant reduction in TC and LGS, non-significant reduction in PM, no change in CP.
1995	Velasco et al.	5 (40%)	Various forms of TC, CP, and AA seizures	CM	65 Hz / 450-800 µA / 0.09 ms	7-33 months	TC seizures decreased, almost disappearing in all patients. Significant reduction in AA. No change in CP.
2000	Velasco et al.	13 (N/A%)	LGS (8) with AA (8), TC (7) and CP (1). FS with secondary generalization (5), CP (4), TC (4), and AA (2)	CM	60 Hz / 4-6 V	±41 months	Mean seizure reduction of 82% in LGS-patients. TC and AA seizures also significantly improved. No significant improvement in CP.
2006	Velasco et al.	13 (N/A%)	LGS with TC (11) and AA (13)	CM	130 Hz / 2-3 V (400-600 µA) / 0.45 ms	±46 months	80% seizure reduction. TC disappeared in 55% of patients and AA disappeared in 27% of patients.
2009	Cukiert et al.	4 (N/A%)	LGS, LLS, and IGE	CM	130 Hz / 2 V / 30 µs	1 year (n = 2)<br>2 years (n = 2)	Seizure frequency reduction of 65-95%.
2013	Valentin et al.	11 (82%)	GE (6) with TC (6) and absence seizures (5). FLE (5) with CP (5) and SPS (1).	CM	60 Hz / up to 5 V / 90 µs	9-month cross-over phase, followed by 5 month ON-phase	>50% reduction in 80% of GE and 40% of FLE patients.
2016	Son et al.	14 (36%)	Multilobar epilepsy (10) and LGS (4)	CM	120-130 Hz / 1-2.6 V / 90-150 µs	±18.2 months	Mean seizure-reduction of 68%. 79% achieved >50% improvement (100% of LGS and 70% of multilobar epilepsy patients).

2017	Kim et al.	10 (40%)	LGS (3) and MFE (7)	CM	130 Hz / 1.5-2.0 V / 90 µs	±21 months	Mean seizure-reduction of 72% in the total patient group, 52% in LGS patients and 80% in MFE patients.
2020	Cukiert et al.	20 (65%)	GE (LGS or LLS)	CM	130 Hz / 3-4.5 V / 300 µs	2.55 years	≥50% reduction in 90% of patients.
2021	Alcala-Zermeno et al.	16 (43%)	Combined GE and FE (7), GE (6), FE (3).	CM CM/ANT	2-100 Hz / 1.0-6.3 V / 60-210 µs	±80 months	Mean significant seizure-reduction of 58%. ≥50% reduction in 63% of patients. No significant difference in CM versus CM/ANT.
Legend*: AA = atypical absence; ANT = anterior nucleus; BL = baseline; CM = centromedian nucleus; CP = complex partial seizures; FE = focal epilepsy FLE = frontal lobe epilepsy; FS = focal seizures; GE = generalized epilepsy; GS = Generalized seizures; IGE = idiopathic generalized epilepsy; LGS = Lennox-Gastaut syndrome; MFE = multifocal epilepsy Myo = myoclonic seizures; PM = partial motor, SPS = simple partial seizure; TA = typical absence seizures; TC = tonic-clonic seizures; TS = tonic seizures; MFE = multifocal epilepsy; MFS = multifocal seizures; N/A = not available.*							

3. STUDIES ON ABLATION/DBS OF THE INTRALAMINAR THALAMUS IN GILLES DE LA TOURETTE

3.1 Ablation (lesion electrode)

Year	Authors	n (% male)	Target	Follow-up	Results
1970	Hassler & Dieckmann	3 (66.7%)	CM-Spv-Voi (2) CM-Spv (1)	N/A	100%, 90%, and 70% reduction in tics.
1973	Hassler & Dieckmann	6 (additional to 1970) (N/A%)	CM-Spv-Voi	N/A	50-100% reduction in tics.
1987	Cappabianca et al.	4 (75%)	IL and MD	> 1 year (2), 2 years (1), several years (1)	Temporary improvement (2), complete regression (1), slight reduction in compulsions (1).

2001	Babel et al.	6 (50%) of 16 (69%)	Various (including LM)	Mean of 12 years (2 lost at follow-up)	Reduction of 61% in vocal tics and 69% in motor tics.
Legend: *CM = centromedian nucleus; IL = intralaminar nucleis; LM = lamella medialis thalamus; MD = medial dorsal nucleus; N/A = not available; Spv = substantia periventricularis; Voi = ventralis-oralis internus.*					

3.2 DBS

Year	Authors	n (% male)	Target	Stimulation parameters	Follow-up	Results
1999	Vandewalle et al.	1 (100%)	CM-Spv-Voi	130 Hz / 1.5 V / 450 µs	1 year	Abolishment of tics.
2003	Visser-Vandewalle et al.	3 (100%)	CM-Spv-Voi	65 or 100 Hz / 2.2-3.0 V / 210 µs	27 months	Mean reduction of 82% in tics.
2006	Ackermans et al.	2 (100%)	CM-Spv-Voi (1), CM-Spv-Voi and GPi stimulation (1)	Patient 1: 130 Hz / 6.4 V / 120 µs<br>Patient 2: 170 Hz / 3.1 V / 210 µs	1 year	Substantial reduction in tics.
2007[10]	Maciunas et al.	5 (100%)	CM-Spv-Voi	130-185 Hz / 3.5 or 3.6 V / 90-210 µs	1 month RCT, 3 months unblinded	3/5 patients experienced a significant reduction in all primary and secondary outcomes (including YGTSS).
2008[11]	Welter et al.	3 (33%)	CM-Pf	130 Hz / 1.5-1.7 V / 60 µs	2 months	45% mean reduction in YGTSS
2008[12]<br>(2012)	Servello et al.<br>(Porta et al.)	18 (83.3%)	CM-Pf-Vo	120-130 Hz / 2.5-4 mV / 90-210 µs	3-18 months, (5-6 year follow-up by Porta et al.)	All patients responded to stimulation. Significant reduction in tic severity, obsessive-compulsive behaviors, anxiety, and depressive symptoms.

2010[13]	Ackermans et al.	2 (100%)	CM-Spv-Voi	Patient 1: 130 Hz / 1.5-1.8 V / 90 µs<br>Patient 2: 100 Hz / 8.5 V / 150 µs	10 years<br>6 years	92.6% improvement in YGTSS,<br>78% improvement in YGTSS.
2011[14]	Ackermans et al.	6 (100%)	CM-Spv-Voi	70-130 Hz / 1.0-7.3 V / 60-210 µs	1 year	49% mean reduction in YGTSS.
2011	Kaido et al.	3 (33.3%)	CM-Pf-Vo	80-130 Hz / 2.1-3.2 V / 180-210 µs	14-21 months	61% mean reduction in YGTSS.
2012	Maling et al.	5 (40%)	CM	100-149.2 Hz / 1-4.50 mA / 80–240 µs	6 months	YGTSS improvements of respectively 1%, 41%, 33%, 32%, and 18%.
2012[15]	Savica et al.	3 (66.7%)	CM-Pf	107-130 Hz / 2.5-4.1 V / 90-120 µs	1 year	70% mean reduction in YGTSS.
2013[16]	Okun et al.	5 (40%)	CM	125 Hz / 1.0-4.5 mA / 80-200 µs	6 months	50% mean reduction in YGTSS.
2016	Testini et al.	11 (73%)	CM-Pf	100 Hz / 2-4 V / 120 us	>2 months	54% mean reduction in YGTSS.
2018	Molina et al.	1 (100%)	CM-Pf	125 Hz / 3.5 mA / 120 µs (RNS)	12 months	48% improvement in YGTSS.
2018	Dowd et al.	13 (N/A %)	CM-Voi	130 Hz / 1-2 V / 90 us	7 years	37% improvement in YGTSS after operation and 50% at last follow-up.
2018	Martinez-Ramirez et al.	93 (78.4%)	CM; vs am-GPi (n=70)	N/A	12 months	46.3% improvement in YGTSS for the CM-group compared to 50.5% in the am-GPi group.
2020	Servello et al.	41 (76%)	CM-Pf/Voi; vs am-GPi (n=14)	N/A	48 months	Improvement of YGTSS in both groups, with better control in psychiatric symptoms in the am-GPi group.
2021	Müller-Vahl et al.	9 (78%)	CM-Voi; vs pvl-GPi	130 Hz / 0.2 V below adverse effects threshold / 210 us	89.9 months	No difference between CM-Voi DBS and pvl-GPi DBS. Various effects of stimulation on individual patients.
2021	Baldermann et al.	8 (75%)	CM-Pf/Voi	N/A	12 months	Various effects, including 44% improvement in YGTSS tic score.
Legend: *CM = centromedian nucleus; CM-Pf = centromedian-parafascicular complex; N/A = not available; Spv = substantia periventricularis; Vo = ventralis-*						

oralis; Voi = ventralis-oralis internus; RNS = responsive neurostimulation; YGTSS = Yale Global Tic Severity Scale; pvl = posteroventral lateral; am = anteromedial; GPi = globus pallidus internus.

4. STUDIES ON ABLATION/DBS OF THE INTRALAMINAR THALAMUS IN DISORDERS OF CONSCIOUSNESS

4.1 Ablation (focused ultrasound)

Year	Authors	n (% male)	Type of DOC	Target	Follow-up	Results
2021	Cain et al.	3 (66%)	MCS	CM-Pf	6 months	1 patient short-term improvement of consciousness, 1 patient improved from MCS- to MCS+ and 1 patient showed no effects.
Legend: *CM-Pf = centromedian-parafascicular complex; MCS = minimally conscious state.*						

4.2 DBS

Year	Autho1rs	n (% male)	Type of DOC	Target	Stimulation parameters	Follow-up	Results
1968	McLardy et al.	1 (100%)	UWS	CM-Pf (as well)	250 Hz / N/A	1 month	EEG modifications, but no effects on consciousness.
1979	Sturm et al.	1 (100%)	UWS	Medial thalamus (as well)	50 Hz / 6-10 V / 0.5 ms	2 months	(Short) improvement in spontaneous movements, verbal contacts and oral feeding.
1984	Cohadon et al.	6 (N/A%)	UWS	CM	50 Hz / 5-10 V / 0.5 ms	2 months DBS (up to 2.5 years follow-up)	3/6 patients no improvements. 3/6 patients return of oral feeding and contact with environment.

1993	Cohadon & Richer	25 (19 new; N/A%)	UWS	CM-Pf	N/A	2 months DBS (1-12 years follow-up)	12/25 patients no effect. 13/25 patients improvement of spontaneous (eye) movements and (verbal) contact with surroundings.
1990/ 2005[17]	Tsubokawa et al./ Yamamoto et al.	26 (N/A%)	UWS (21) MCS (5)	CM-Pf (24) and nucleus cuneiformis (2)	25 Hz / N/A	±10 years	13/21 UWS patients no effect. 8/21 UWS patients recovery of oral feeding en (verbal) contact with surroundings. 4/5 MCS patients improvement of (verbal) contact with surroundings and emergence of bedridden state.
2007	Schiff et al.	1 (100%)	MCS	CL	100 Hz / 4 V / N/A	6 months cross-over (follow-up >1 year after surgery)	Long-term return of arousal/awareness, oral feeding, speech/verbal contact and CRS-improvements (subscales).
2016	Magrassi et al.	3 (100%)	UWS (1) MCS (2)	CM-Pf	80-110 Hz / N/A	18-60 months	No return of full consciousness, though small improvements in CRS-scores (change of 2.33-3 at the end of follow-up).
2016[18]	Adams et al.	1 (100%)	MCS	CL	N/A	60 months	No significant change in CRS-R. Change in sleep dynamics.
2018	Chudy et al.	14 (64%)	UWS (10) MCS (4)	CM-Pf	25 Hz / 2.5-3.5 V / 90 µs	38-60 months	2/4 MCS patients regained consciousness/independence. 1/4 MCS patients regained consciousness, but still in wheelchair. 1/10 UWS patients showed some signs of consciousness. 3 patients died and rest (7/14) showed no improvement.
2018	Lemaire et al.	5 (40%)	UWS (1) MCS (4)	CM-Pf	30 Hz / 4-6 V / 60 µs	11 months	1/1 UWS and 1/4 MCS patients showed a slight increase in mean CRS-R score.

Legend: *CL = central lateral nucleus; CM = centromedian nucleus; CM-Pf = centromedian-parafascicular complex; EEG = Electroencephalography; CRS-R = coma recovery scale-revised; MCS = minimally conscious state; N/A = not available; UWS = unresponsive wakefulness syndrome.*

5. STUDIES ON ABLATION/DBS OF THE INTRALAMINAR THALAMUS IN MOVEMENT DISORDERS

5.1 Ablation (lesion electrode)

Year	Authors	Type	n (% male)	Target	Follow-up	Results
1963	Markham & Rand	PD	9 (N/A%)	Centromedian thalamus	6 months - 5 years	N/A (not specified).
1965	Adams & Rutkin	PD (unilateral/bilateral tremor)	26 (62%)	Centromedian thalamus	Variable (on average >3 months)	Majority of patients with unilateral disease have excellent (free of tremor) or good result (mild tremor). Majority of patients with bilateral disease obtained good results (mild tremor).
1984	Vasin et al.	PD (severe akinesia)	15 (N/A%)	Centromedian thalamus	N/A	3/15 marked improvement of motor/speech function. 5/15 minor improvements of motor function. 2/15 no effects. 2/15 increase of akinesia. 3/15 no ablation (after stimulation).
1996	Jeanmonod et al.	Various (including Parkinson/ cerebellar tremor)	22 (N/A%)	Medial thalamus	±18 months	50-100% relief of symptoms as tremor, rigidity, and bradykinesia in 43% of patients, but limited decrease in overall mean UPDRS (mean change from 47 to 37).
Legend: *N/A = not available; PD = Parkinson's Disease; UPDRS = Unified Parkinson's Disease Rating Scale.*						

5.2 DBS

Year	Authors	Type	n (% male)	Target	Stimulation parameters (reported)	Follow-up	Results

1980	Andy et al.	PD, spasticity, and (cervical) dystonia (in patients with pain)	4 (25%)	CM-Pf	125 Hz / 7-8 V / 0.5 ms and 75 Hz / 4 V / 0.4 ms	N/A	Variable stimulation induced reduction of tremor, rigidity, and dyskinesia.
2002	Krauss et al.	Tremor/dyskinesia (in patients with pain)	3 (66%)	CM-Pf	130 Hz / Variable amplitude / 180-210 µs	0, 1 and 2 years	Considerable improvement of tremor/dyskinesia in 2/3 patients.
2006	Mazzone et al.	PD	6 (N/A%)	CM-Pf	185 Hz / 1.5-2.5 V / 90 µs	N/A	Slightly effective in reducing rigidity and akinesia, but more effect on freezing (significantly better when combined with regular GPi DBS).
2008	Peppe et al.	PD	2 (0%)	CM-Pf	185 Hz / 1.8-3.1 V (monopolar) or 2-3 V (bipolar)	N/A	Very effective in reducing hand tremor.
2009	Stefani et al. (follow-up of Mazzone et al.)	PD	8 (13 total, N/A%)	CM-Pf	185 Hz / 1.5–2.5 V (monopolar) or 2-3 V (bipolar) / 90 µs	Varies (8 patients >3 years)	Variable stimulation induced reduction in tremor and dyskinesia (next to regular STN or GPi DBS).

Legend: *CM-Pf = centromedian-parafascicular complex; GPi = internal globus pallidus; PD = Parkinson's disease; STN = subthalamic nucleus; N/A = not available.*

SUPPLEMENTAL REFERENCES (NOT IN MAIN MANUSCRIPT)

1. Niizuma, H., Kwak, R., Ikeda, S., Ohyama, H., Suzuki, J. and Saso, S. (1982). Follow-up Results of Centromedian Thalamotomy for Central Pain. Appl Neurophysiol 45, 324-325.

2. Young, R.F. (1996). Functional neurosurgery with the Leksell Gamma Knife. Stereot Funct Neuros 66, 19-23.

3. Jeanmonod, D., Magnin, M., Morel, A. and Siegemund, M. (2001). Surgical control of the human thalamocortical dysrhythmia: I. Central lateral thalamotomy in neurogenic pain. Thalamus & Related Systems 1, 71-79.

4. Boethius, J., Lindblom, U., Meyerson, B. and Widen, L. (1976). Effects of multifocal brain stimulation on pain and somatosensory functions. In: *Sensory Functions of the Skin in Primates*. Elsevier, pps. 531-548.

5. Thoden, U., Doerr, M., Dieckmann, G. and Krainick, J.U. (1979). Medial Thalamic Permanent Electrodes for Pain Control in Man - Electro-Physiological and Clinical-Study. Electroen Clin Neuro 47, 582-591.

6. Andy, O.J. (1983). Thalamic-Stimulation for Chronic Pain. Appl Neurophysiol 46, 116-123.

7. Krauss, J., Pohle, T., Weigel, R. and Kalbarczyk, A. (2001). Somatosensory thalamic stimulation versus center median-parafascicular complex stimulation in 11 patients with neuropathic pain. Stereotact Funct Neurosurg 77, 194.

8. Hollingworth, M., Sims-Williams, H.P., Pickering, A.E., Barua, N. and Patel, N.K. (2017). Single Electrode Deep Brain Stimulation with Dual Targeting at Dual Frequency for the Treatment of Chronic Pain: A Case Series and Review of the Literature. Brain Sci 7.

9. Velasco, F., Velasco, M., Ogarrio, C. and Fanghanel, G. (1987). Electrical-Stimulation of the Centromedian Thalamic Nucleus in the Treatment of Convulsive Seizures - a Preliminary-Report. Epilepsia 28, 421-430.

10. Maciunas, R.J., Maddux, B.N., Riley, D.E., Whitney, C.M., Schoenberg, M.R., Ogrocki, P.J., Albert, J.M. and Gould, D.J. (2007). Prospective randomized double-blind trial of bilateral thalamic deep brain stimulation in adults with Tourette syndrome. J Neurosurg 107, 1004-1014.

11. Welter, M.L., Mallet, L., Houeto, J.L., Karachi, C., Czernecki, V., Cornu, P., Navarro, S., Pidoux, B., Dormont, D., Bardinet, E., Yelnik, J., Damier, P. and Agid, Y. (2008). Internal pallidal and thalamic stimulation in patients with Tourette syndrome. Arch Neurol-Chicago 65, 952-957.

12. Servello, D., Porta, M., Sassi, M., Brambilla, A. and Robertson, M.M. (2008). Deep brain stimulation in 18 patients with severe Gilles de la Tourette syndrome refractory to treatment: the surgery and stimulation. J Neurol Neurosur Ps 79, 136-142.

13. Ackermans, L., Duits, A., Temel, Y., Winogrodzka, A., Peeters, F., Beuls, E.A.M. and Visser-Vandewalle, V. (2010). Long-term outcome of thalamic deep brain stimulation in two patients with Tourette syndrome. J Neurol Neurosur Ps 81, 1068-1072.

14. Ackermans, L., Duits, A., van der Linden, C., Tijssen, M., Schruers, K., Temel, Y., Kleijer, M., Nederveen, P., Bruggeman, R., Tromp, S., van Kranen-Mastenbroek, V., Kingma, H., Cath, D. and Visser-Vandewalle, V. (2011). Double-blind clinical trial of thalamic stimulation in patients with Tourette syndrome. Brain 134, 832-844.

15. Savica, R., Stead, M., Mack, K.J., Lee, K.H. and Klassen, B.T. (2012). Deep Brain Stimulation in Tourette Syndrome: A Description of 3 Patients With Excellent Outcome. Mayo Clin Proc 87, 59-62.

16. Okun, M.S., Foote, K.D., Wu, S.S., Ward, H.E., Bowers, D., Rodriguez, R.L., Malaty, I.A., Goodman, W.K., Gilbert, D.M., Walker, H.C., Mink, J.W., Merritt, S., Morishita, T. and Sanchez, J.C. (2013). A Trial of Scheduled Deep Brain Stimulation for Tourette Syndrome Moving Away From Continuous Deep Brain Stimulation Paradigms. Jama Neurol 70, 85-94.

17. Yamamoto, T., Kobayashi, K., Kasai, M., Oshima, H., Fukaya, C. and Katayama, Y. (2005). DBS therapy for the vegetative state and minimally conscious state. In: *Re-Engineering of the Damaged Brain and Spinal Cord*. Springer, pps. 101-104.

18. Adams, Z.M., Forgacs, P.B., Conte, M.M., Nauvel, T.J., Drover, J.D. and Schiff, N.D. (2016). Late and progressive alterations of sleep dynamics following central thalamic deep brain stimulation (CT-DBS) in chronic minimally conscious state. Clin Neurophysiol 127, 3086-3092.
